# The association between loneliness and psychiatric symptomatology in older psychiatric outpatients

**DOI:** 10.1192/j.eurpsy.2023.134

**Published:** 2023-07-19

**Authors:** N. Schutter

**Affiliations:** geriatric psychiatry, Arkin Mental Health Organization, Amsterdam, Netherlands

## Abstract

**Abstract:**

Purpose: loneliness in adults increases with age. Although loneliness has been found to be associated with psychiatric disorders and dementia, no information is available on prevalence of loneliness in older psychiatric patients. Given the negative consequences of loneliness for morbidity and mortality, identification of specific populations vulnerable to loneliness is important. The aims of the present research were to examine prevalence of loneliness in older psychiatric outpatients, including gender differences and associations with psychiatric disorders and with social situation.

**Methods:**

interviews were done in 181 patients from an outpatient clinic for geriatric psychiatry between September 2013 and February 2018, using questionnaires regarding loneliness, depression, anxiety, frailty and alcohol use.

**Results:**

prevalence of loneliness was as high as 80%. Loneliness was associated with having less social contacts, in women only. There were no associations with DSM-IV-TR-classifications. However, loneliness was associated with higher scores on a depression questionnaire. There were no significant differences in intensity of treatment between lonely and non-lonely participants.

**Conclusion:**

Loneliness is highly prevalent in older psychiatric outpatients, with men and women equally affected. Loneliness should be assessed in all older psychiatric patients, especially when they show high scores on symptom checklists or have a restricted social network.

**Reference:**

Schutter et al. (2022) The association between loneliness and psychiatric symptomatology in older psychiatric outpatients. *Journal of Geriatric Psychiatry and Neurology* 35: 778-788.

**Disclosure of Interest:**

None Declared

